# Supplementary material for: A new class of cyclin dependent kinase in Chlamydomonas is required for coupling cell size to cell division
Source: eLife. 2016 Mar 25;5:e10767. doi: 10.7554/eLife.10767 (PMC4841777; doi:10.7554/eLife.10767)
Supplement: Supplementary file 1. — DOI: http://dx.doi.org/10.7554/eLife.10767.021 [file elife-10767-supp1.docx]

**Supplementary file 1. CDK sequences used for phylogenetic analysis**

| **CDK** | **Genbank Accession** |
| --- | --- |
| atCDKA1 | NP_566911 |
| atCDKB1 | NP_190986 |
| atCDKB2 | NP_177780 |
| crCDKA1 | XP_001698637 |
| crCDKB1 | XP_001701299 |
| crCDKG1 | XP_001696492 |
| crCDKG2 | XP_001701126 |
| drCDK1 | NP_997729 |
| drCDK2 | NP_998571 |
| dmCDK4 | NP_725594 |
| hsCDK1 | NP_001777 |
| hsCDK2 | CAA43985 |
| hsCDK3 | NP_001249 |
| hsCDK4 | CAG47043 |
| hsCDK6 | NP_001138778 |
| mmCDK2 | NP_904326 |
| rnCDK4 | AAA40903 |
| rnCDK6 | NP_001178790 |
| spCDK2 | XP_001178731 |
| spCDK4 | NP_999689 |
| vcCDKG1 | XP_002946192 |
