# Supplementary material for: A new class of cyclin dependent kinase in Chlamydomonas is required for coupling cell size to cell division
Source: eLife. 2016 Mar 25;5:e10767. doi: 10.7554/eLife.10767 (PMC4841777; doi:10.7554/eLife.10767)
Supplement: Supplementary file 2. — DOI: http://dx.doi.org/10.7554/eLife.10767.022 [file elife-10767-supp2.docx]

**Supplementary file 2. Primers used in this study**

| ***NIT1 insertion mapping primers*** |  | | | |  | | | |
| --- | --- | --- | --- | --- | --- | --- | --- | --- |
| **Name** | **Sequence (5' to 3')** | | | |  | | | |
| IMP NIT56 3-4 | GTTTAGCGCATGTCCTTGATA | | | |  | | | |
| IMP NIT56 3-3 | CGGGCTGCGAGAGGAGTTTA | | | |  | | | |
| IMP NIT56 3-2 | ATCGCTAGAGATTGTGGCCA | | | |  | | | |
| IMP NIT56 3-1 | GCTTTGGCTTCGCCTTCCGT | | | |  | | | |
| IMP NIT56 5-4 | atggttgctttgacgtatgcg | | | |  | | | |
| IMP NIT56 5-3 | actaaatcggaaccctaaagg | | | |  | | | |
| IMP NIT56 5-2 | aaaaccgtctatcagggcga | | | |  | | | |
| IMP NIT56 5-1 | gaaatcggcaaaatcccttat | | | |  | | | |
| Adaptor plus strand | GTAATACGACTCACTATAGAGTACGCGTGGTCGACGGCCCGGGCTGGT | | | |  | | | |
| Adaptor minus strand | ACCAGCCCGG | | | |  | | | |
| AP1 | GTAATACGACTCACTATAGAGT | | | | Adaptor primers | | | |
| AP2 | ACTATAGAGTACGCGTGGT | | | | Adaptor primers | | | |
| ***Primers for 7kb CDKG1 genomic DNA fragment cloning***  **Name** | **Sequence (5’ to 3’)** | | | |  | | |  |
| OER0003 | CCGTCTAGATAGCGGGGTTGTAACCGTGGA | | | |  | | |  |
| OER0004 | CCGGAATTCGCTGCACGCTGGACTTGCTA | | | |  |  |  |  |
| ***Genotyping primers shown in Figure 1-figure supplement 1***  **Name** | **Sequence (5’ to 3’)** | **PCR condition (w/ Taq DNA polymerase)** | | | | **PCR Product** | | |
| CDKG1 8F (**a1**) | TGTCGGTCTGGACCTGCTAC | 94°C 3 min; 94°C 30s-60°C 30s-72°C 30s (32 cycles); 72°C 5 min | | | | *CDKG1* last exon with adjacent 3’UTR region | | |
| CDKG1 3’-Rev-2 (**b1**) | ATGTGGGGATTCAGCCGTAG |  |  |  |  |  |  |  |
| IMP NIT56 5-2 (**a4**) | aaaaccgtctatcagggcga | 94°C 3 min; 94°C 30s-55°C 30s-72°C 30s (32 cycles); 72°C 5 min | | | | *NIT* and *CDKG1* 3’ UTR junction | | |
| CDKG1 3’-Rev-2 (**b1**) | ATGTGGGGATTCAGCCGTAG |  |  |  |  |  |  |  |
| ***Primers for CDKG1 site directed mutagenesis***  **Name** | **Sequence (5’ to 3’)** | | | **Mutation** | | | | |
|  |  | | |  | | | | |
| CDKG1 KD-forward | ATGAGCTGCTGGCCATCCGCAAGGTGGTACACAGCATT | | | CDKG1 kinase dead mutation | | | | |
| CDKG1 KD-reverse | AATGCTGTGTACCACCTTGCGGATGGCCAGCAGCTCAT | | |  |  |  |  |  |
| CDKG1 T-loop-forward | CCAACGAGGACCGCGCCTACGCCGAGCGTGTG | | | T-loop inactive mutation | | | | |
| CDKG1 T-loop-reverse | CACACGCTCGGCGTAGGCGCGGTCCTCGTTGG | | |  |  |  |  |  |
|  | | | |  | | | | |
| ***Primers for RT-PCR***  **Name** | **Sequence (5’ to 3’)** | | | **PCR condition (w/ Taq DNA polymerase)** | | | | |
| 18SrRNA-1 | ATCTGCGAAAGCATTTGCCA | | | 94°C 3min; 94°C 30s-60°C 30s-72°C 20s (13-20 cycles); 72°C 7min | | | | |
| 18SrRNA-2 | CGGCATCGTTTATGGTTGAGAC | | |  |  |  |  |  |
| GBLP-1 | GAGTCCAACTACGGCTACGC | | | 94°C 3min; 94°C 30s-60°C 30s-72°C 30s (15-20 cycles); 72°C 7min | | | | |
| GBLP-2 | AGCTTGCAGTTGGTCAGGTT | | |  |  |  |  |  |
| CYCD1 A-1F | CGGAGGATGTCCTAGACGAG | | | 94°C 3min; 94°C 30s-55°C 30s-72°C 30s (28-34 cycles); 72°C 7min | | | | |
| CYCD1 A-1R | CAGGTCTTGCCTGATCACAA | | |  |  |  |  |  |
| CYCD2 F664 | GTACCCAACTCGCTGGTGTT | | | 94°C 3min; 94°C 30s-58°C 30s-72°C 30s (28-34 cycles); 72°C 7min | | | | |
| CYCD2 R826 | TGTAGCCGTAAGGCAGGAAG | | |  |  |  |  |  |
| CYCD3 F228 | CGAGTCGTCATACTGCTGGA | | | 94°C 3min; 94°C 30s-58°C 30s-72°C 30s (28-32 cycles); 72°C 7min | | | | |
| CYCD3 R399 | ATAGGACGTGCTCACGAAGG | | |  |  |  |  |  |
| CYCD4 3F | GTCCTTGGCCATGAAGTACG | | | 94°C 3min; 94°C 30s-52°C 30s-72°C 30s (30-34 cycles); 72°C 7min | | | | |
| CYCD4 2R | TCAGCAAGCGAGCACAGCA | | |  |  |  |  |  |
| CDKG1 1F (**a3** in Figure1-figure supplement 1C) | ATGGCTCCTGGCTTTGGCAAC | | | 94°C 3min; 94°C 30s-60°C 30s-72°C 20s (20-28 cycles); 72°C 7min | | | | |
| CDKG1 10R (**b2** in Figure1-figure supplement 1C) | GCGAATTCGTACTTCTGAAGCGCGCC | | |  |  |  |  |  |
| 3xHA 1F (**a2** in Figure1-figure supplement 1C) | ACTAGTATGTCTAGTTACCCATACGATGTTCC | | | 94°C 3min; 94°C 30s-58°C 30s-72°C 20s (20-30 cycles); 72°C 7min | | | | |
| CDKG1 10R (**b2** in Figure1-figure supplement 1C ) | GCGAATTCGTACTTCTGAAGCGCGCC | | |  |  |  |  |  |
| CDKG1 RT-1F  (for RT-PCR in Figure 3-figure supplement 1B) | CTGAGGCAGTTCAAGGCTCT | | | 94°C 3min; 94°C 30s-60°C 30s-72°C 20s (20-28 cycles); 72°C 7min | | | | |
| CDKG1 RT-1R  (for RT-PCR in Figure 3-figure supplement 1B ) | CGGGATACCAGTGGAACTTG | | |  |  |  |  |  |
| CDKB1-3  (for RT-PCR in Figure 3-figure supplement 1B ) | GACAACGCTGCGTGAGATTTC | | | 94°C 3min; 94°C 30s-60°C 30s-72°C 20s (20-28 cycles); 72°C 7min | | | | |
| CDKB1-4  (for RT-PCR in Figure 3-figure supplement 1B ) | ACCAGGTAAAGGCATGGCTTG | | |  |  |  |  |  |
| CDKG1 8F (For RT-PCR in Fig. 2C) | TGTCGGTCTGGACCTGCTAC | | | 94°C 3min; 94°C 30s-55°C 30s-72°C 30s (32-38 cycles); 72°C 7min | | | | |
| pSAD 3'UTR (For RT-PCR in Fig. 2C) | CAGTCACGCTGTCTCCCCCTGTC | | |  |  |  |  |  |
| ***Primers used to insert 3xHA tag in CDKG1 genomic DNA by overlapping PCR***  **Name** | **Sequence (5’ to 3’)**  **(highlight overlapping regions)** | | | **PCR condition (w/ Phusion® DNA polymerase)** | | | | **PCR product** |
| gG1 1F (SpeI) | ACACTAGTGTTGGGTACCACGGAGAG | | | 96°C 3 min; 96°C 15s-60°C 30s-72°C 20s (30 cycles); 72°C 5 min | | | | 3xHA-gCDKG1 overlapping fragment 1 |
| gG1-HA 1R | gtatgggtaactagacatAGCAGCCAGTCTTCAAAGT | | |  |  |  |  |  |
| gG1-HA 2F-2 | AACTTTGAAGACTGGCTGCTatgtctagttacccatacgatgttccg | | | 96°C 3 min; 96°C 15s-60°C 30s-72°C 20s (30 cycles); 72°C 5 min | | | | 3xHA-gCDKG1 overlapping fragment 2 |
| gG1-HA 2R-2 | CGCGGTGGCAAAGTTGCCAAAGCCAGGAGCtctagtagcgtaatctggaaCgtc | | |  |  |  |  |  |
| gG1-HA 3F | ttccagattacgctactagaGCTCCTGGCTTTGGCAACTTTGCCACCGCG | | | 96°C 3 min; 96°C 15s-60°C 30s-72C 30s (30 cycles); 72°C 5 min | | | | 3xHA-gCDKG1 overlapping fragment 3 |
| gG1 3R (Bgl II) | TCCAGATCTGGCCATGACCACCCAG | | |  |  |  |  |  |
| ***Primers for cDNA cloning and plasmid construction***  ***Name*** | **Sequence (5’ to 3’)** | | **PCR condition (w/ Phusion® DNA polymerase)** | | | | **Plasmid generated** | |
| CYCA1 2F (NdeI) | catATGAGCTCTCGCGTCGGCT | | 96°C 1min; 96°C 15s-60°C 20s-72°C 30s (30 cycles); 72°C 5min | | | | pGAD-CYCA1;  pGBK-CYCA1 | |
| CYCA1 2R (EcoRI) | gaattcTCACGAGCGGTGAATGCCGctg | |  |  |  |  |  |  |
| CYCB1 2F (NdeI) | catATGGCTCTTCGTGCGGTT | | 96°C 1min; 96°C 15s-55°C 20s-72°C 30s (30 cycles); 72°C 5min | | | | pGAD-CYCB1;  pGBK-CYCB1 | |
| CYCB1 2R (EcoRI) | gaattcTTAAGCCACTGGCTGCGC | |  |  |  |  |  |  |
| CYCD2 2F (NdeI) | catATGGCTCCGTTTGGTCCCA | | 96°C 1min; 96°C 15s-60°C 30s-72°C 30s (30 cycles); 72°C 5min | | | | pGAD-CYCD2;  pGBK-CYCD2 | |
| CYCD2 2R (EcoRI) | gaattcTCACCGCTGAGCGGACGG | |  |  |  |  |  |  |
| CYCD3 2F (NdeI) | catATGGAGTGCTCGAGCCCCT | | 96°C 1min; 96°C 15s-58°C 30s-72°C 30s (30 cycles); 72°C 5min | | | | pGAD-CYCD3;  pGBK-CYCD3;  pGAD-CYCD3^AxAxA^ | |
| CYCD3 2R (EcoRI) | gaattcCTACTGCTGCACCGGCTG | |  |  |  |  |  |  |
| CDKA1 2F (XhoI) | ccgctcgagATGGACAAGTATGAGAAGCTAGAG | | 96°C 1min; 96°C 15s-58°C 30s-72°C 30s (30 cycles); 72°C 5min | | | | pGAD-CDKA1;  pGBK-CDKA1 | |
| CDKA1 2R (BamHI) | ggatccTCAGCGCATCACGCCCATT | |  |  |  |  |  |  |
| CDKB1 2F (NdeI) | catATGGATGCCTACGAGAAGATTGAG | | 96°C 1min; 96°C 15s-55°C 30s-72°C 30s (30 cycles); 72°C 5min | | | | pGAD-CDKB1;  pGBK-CDKB1 | |
| CDKB1 2R (EcoRI) | gaattcTCAGCAGACAACGTTGGCGG | |  |  |  |  |  |  |
| CDKG1 2F (NdeI) | CATatgGCTCCTGGCTTTGGCAAC | | 96°C 1min; 96°C 15s-60°C 30s-72°C 30s (30 cycles); 72°C 5min | | | | pGAD-CDKG1;  pGBK-CDKG1;  pEZ-CDKG1;  pEZ-CDKG1^kd^;  pET28a-CDKG1 | |
| CDKG1 3R (EcoRI) | GGAATTCTCACACGACGACGTCGTTGA | |  |  |  |  |  |  |
| CDKG1 4F (XhoI) | ccgctcgagATGGCTCCTGGCTTTGGCAA | | 96°C 1min; 96°C 15s-60°C 30s-72°C 30s; (30 cycles); 72°C 5min | | | | pVL399-CDKG1;  pVL399-CDKG1^kd^;  pVL399-CDKG1^ΔT-LOOP^ | |
| CDKG1 2R (XhoI) | ctcgagctacacgacgacgtcgttga | |  |  |  |  |  |  |
| CDKG1 TF2 (NdeI) | ccgcatatgAAGTACATCAAGATCGGTCAGCTGT | | 96°C 1min; 96°C 15s-60°C 30s-72°C 20s; (28 cycles); 72°C 5min | | | | pGAD-CDKG1-C;  pGBK-CKG1-C | |
| CDKG1 3R (EcoRI) | GGAATTCTCACACGACGACGTCGTTGA | |  |  |  |  |  |  |
| CDKG1 2F (NdeI) | CATatgGCTCCTGGCTTTGGCAAC | | 96°C 1min; 96°C 10s-60°C 15s-72°C 10s (25 cycles); 72°C 5min | | | | pET28a-CDKG1-N | |
| CDKG1 10R-2(EcoRI) | GCGAATTCGTACTTCTGAAGCGCGCC | |  |  |  |  |  |  |
| gCDKG1 pro 1F (XbaI) | CCGTCTAGATAGCGGGGTTGTAACCGTGGA | | 96°C 2min; 96°C 10s-58°C 30s-72°C 30" (36 cycles); 72°C 5min | | | | pCDKG1-luc-G1 3’UTR;  pCDKG1-luc-RBCS | |
| gCDKG1 pro 1R (XhoI) | CCGCTCGAGAGCAGCCAGTCTTCAAAGTTCAA | |  |  |  |  |  |  |
| CDKG1 3’UTR fw (Bgl II) | AGATCTGCGTGCTGCTTGCTCGCC | | 96°C 2min; 96°C 10s-55°C 30s-72°C 1’ (36 cycles); 72°C 5min | | | | pCDKG1-luc-G1 3’UTR;  pHsp70A/Rbcs2-luc-G1 3’UTR | |
| CDKG1 3’UTR rv (NcoI) | CCATGGTACTGTGAGTGTTCCTGCTGA | |  |  |  |  |  |  |
| SalI 3HA F | CGCGTCGACCATATGTCTAGTTACCCATACG | | 96°C 1min; 96°C 15s-60°C 30s-72°C 30s (30 cycles); 72°C 5min | | | | pL14-HA-CDKG1 & pL14-HA-CDKG1 kinase dead (K125R) | |
| BclI CDKG1 R | CCGTGATCATCACACGACGACGTCGTTG | |  |  |  |  |  |  |
| SalI CDKG1 F | CGCGTCGACATGGCTCCTGGCTTTGGCAAC | | 96°C 1min; 96°C 15s-60°C 30s-72°C 30s (30 cycles); 72°C 5min | | | | pL14-CDKG1 & pL14-CDKG1 kinase dead (K125R) | |
| BclI CDKG1 R | CCGTGATCATCACACGACGACGTCGTTG | |  |  |  |  |  |  |
| ***Primers for qRT-PCR***  ***Name*** | ***Sequence (5’ to 3’)*** | | ***Target gene*** | | | | ***qPCR conditions*** | |
| GBLP-3 | GTCATCCACTGCCTGTGCTTCT | | *GBLP* | | | | 96°C 3min; 96°C 10s-62°C 10s-72°C 30”(39 cycles) | |
| GBLP-4 | GGCCTTCTTGCTGGTGATGTT | |  |  |  |  |  |  |
| LucF | CTGCCTGTCCCACATCAAGT | | Luciferase | | | |  |  |
| LucR | ATGAACTGCTCCATCGGCTC | |  |  |  |  |  |  |
| CDKG1 RT-3F | CGTGTGGTGACGCTGTACTA | | *CDKG1* | | | |  |  |
| CDKG1 RT-3R | GGAACAGAAGCCCGATCTC | |  |  |  |  |  |  |
